# A Comparative Study on the Dynamic EEG Center of Mass with Different References

**DOI:** 10.3389/fnins.2017.00509

**Published:** 2017-09-12

**Authors:** Yun Qin, Xiuwei Xin, Hao Zhu, Fali Li, Hongchuan Xiong, Tao Zhang, Yongxiu Lai

**Affiliations:** ^1^Key Laboratory for NeuroInformation of Ministry of Education, Center for Information in Medicine, University of Electronic Science and Technology of China Chengdu, China; ^2^High-Field Magnetic Resonance Brain Imaging Key Laboratory of Sichuan Province, School of Life Science and Technology, University of Electronic Science and Technology of China Chengdu, China

**Keywords:** neutral reference, center of mass, ERPs, trajectory, traveling velocity

## Abstract

One of the most fundamental issues during an EEG study is choosing an available neutral reference. The infinity zero reference obtained by the reference electrode standardization technique (REST) has been recommended and used for its higher accuracy. This paper examined three traditional references, the average reference (AR), the linked mastoids reference (LM), and REST, in the study of the EEG center of mass (CM) using simulated and real ERPs. In the simulation, the relative error of REST was the smallest among the references. As for the ERP data with the visual oddball paradigm, the dynamic CM trajectory and its traveling velocity obtained by REST characterized three typical stages in spatial domain and temporal speed metrics, which provided useful information in addition to the distinct ERP waveform in the temporal domain. The results showed that the CM traveling from the frontal to parietal areas corresponding to the earlier positive components (i.e., P200 and P250), stays temporarily at the parietal area corresponding to P300 and then returns to the frontal area during the recovery stage. Compared with REST, AR, and LM not only changed the amplitude of P300 significantly but distorted the CM trajectory and its instantaneous velocity. As REST continues to provide objective results, we recommend that REST be used in future EEG/ERP CM studies.

## Introduction

Electroencephalogram (EEG) denotes the spatiotemporal dynamic process of the encephalic neural activities. The choice of reference influences both the spatial and temporal aspects of the EEG, which is one of the most fundamental issues in EEG analysis and interpretation. For the scalp distribution of the EEG at a set time point, different reference choice will introduce a constant value at all locations, similar to raising or lowering the water level of a landscape, without changing the shape (Pascualmarqui and Lehmann, 1993; Geselowitz, 1998). When the reference site on the body surface is active, the EEG dynamic process may be distorted due to the temporal bias of the reference signal. Thus, research teams are searching for the best available reference option for cross-study comparison (Kayser and Tenke, 2010; Nunez, 2010).

Different reference methods, such as, the average reference (AR), the linked mastoids reference (LM), and the vertex reference (CZ), are commonly used under certain assumptions (Yao, 2001; Nunez and Srinivasan, 2006a; Marzetti et al., 2007) while ignoring that they are not zero references. Previous studies have found that these references can introduce false voltage waveform fluctuation, spectrum scalp distribution shifts, and EEG network distortion (Yao, 2001; Kayser and Tenke, 2010; Nunez, 2010; Qin et al., 2010; Tian and Yao, 2013; Liu et al., 2015; Chella et al., 2016). AR is limited by an insufficient spatial sampling of the scalp field, as well as by the source distribution, which brings potential variance and network distortion (Yao, 2001; Zhai and Yao, 2005; Qin et al., 2010; Tian and Yao, 2013). The LM reference, obtained by combining the two mastoid electrodes, is independent of the electrode montage; however, the LM reference is challenged by its effect on the bilateral and posterior electrodes resulting in the power shift to the frontal and superficial positions (Yao et al., 2005). Although the Cz electrode is located farther from the sources, but the information-contained potential of the Cz electrode introduces large error to the vertex areas (Liu et al., 2015).

The reference electrode standardization technique (REST) proposed by Yao has been increasingly used in EEG studies (Yao, 2001). REST provides a standardized technique for approximately transforming multi-channel recordings with a scalp point into real EEG data using an infinity neutral reference. Studies on spectra imaging, EEG coherence, and connectivity using spontaneous EEG showed that REST tends to obtain more accurate and objective results (Yao et al., 2005; Marzetti et al., 2007; Qin et al., 2010). ERP components, cognitive psychology (Yao et al., 2007; Tian and Yao, 2013; Liu et al., 2015), and clinical EEG analysis (Xu et al., 2014) have shown that REST is also valuable in cognition and disease recognition.

Center of mass (CM) has been used as a metric to investigate the systematic integration and variability of the EEG (Wackermann et al., 1993; Manjarrez et al., 2007). In physics, CM is the point in an object or system that can describe the system's response to external forces and torques. Computationally, CM is the average of the masses factored by their distances from a reference point. For EEG study, CM is usually calculated by averaging the scalp potentials weighted by the spatial coordinates of all channels. Thus, spatial information from the entire scalp is used to calculate the CM rather than choosing only certain electrodes. The micro-states of the EEG time series can be distinguished by analyzing the positive and negative CM of the EEG (Wackermann et al., 1993). By applying the CM method, Manjarrez et al. found that the alpha wave trajectory starts and ends in specific brain regions (Manjarrez et al., 2007). Compared to the traditional focal electrodes and topographical analysis, CM trajectory is useful for presenting global temporal and spatial properties. In addition, varying the CM's instantaneous velocity may provide quantitative information on EEG patterns with certain cognitive characteristics.

In this study, we comparatively investigated the dynamic CM of the EEG to evaluate the performance of different reference strategies. Simulation was conducted to test the accuracy of EEG references. ERP data from the visual oddball paradigm were then used to show the dynamic CM trajectory with different references. In addition, the CM's traveling velocity was computed to helpfully reveal the P300 cognitive mechanism.

## Materials and methods

### Reference electrode standardization technique (REST)

REST is derived from the theoretical relationship between the scalp recordings with a body reference point and a distributed source model *S* (Yao, 2001; Yao et al., 2005). For a neutral reference at infinity, we have:

(1) $$\begin{array}{l} V=GS \end{array}$$

where the lead field matrix *G* depends on the head model, source configuration and electrode montage and has a reference at infinity. Similarly, the scalp EEG recordings *V*_*cz*_ referenced to CZ can be generated by:

(2) $$\begin{array}{l} V_{CZ}=G_{CZ}S \end{array}$$

where *G*_*cz*_ is the EEG lead-field matrix with the CZ reference. A minimum norm solution (MNS) for the source distribution *S* is given by:

(3) $$\begin{array}{l} S={G_{CZ}}^{-}V_{CZ} \end{array}$$

where ${G_{CZ}}^{-}$ denotes the Moore-Penrose generalized inverse of the matrix *G*_*cz*._

From Equations (2) and (3), we can see that the source *S* is the same, which indicates that the source localization and activity will not be influenced by the references. Thus, the potential with a reference at infinity can be reconstructed as the following:

(4) $$\begin{array}{l} V_{IR}=G\left({G_{CZ}}^{-}V_{CZ}\right)=UV_{CZ},U=GG_{CZ}- \end{array}$$

where U is the final transfer matrix determined by the lead-field matrices *G* and *G*_*cz*_, both of which can be easily derived. Details of the REST algorithm can be found in Yao (2001), and the free software can be downloaded at www.neuro.uestc.edu.cn. Similarly, recordings with linked mastoids and average references (Dien, 1998; Hagemann et al., 2001) can be transformed to the neutral infinite reference using the formula in Equation (4) and different lead field matrix is used for the chosen reference.

In this study, the head model for all cases was a three-concentric-sphere model, and the normalized radii of the three concentric spheres were 0.87 (inner radius of the skull), 0.92 (outer radius of the skull), and 1.0 (radius of the scalp). The conductivities were 1.0, 0.0125, and 1.0 for the brain, skull and scalp, respectively. The center of the spheres was defined as the coordinate origin. The x-axis was oriented from the origin to the right ear, and the y-axis was oriented from the origin to the nasion. The z-axis was oriented from the origin to the vertex.

### The center of mass (CM)

In this study, we used the positive CM to evaluate the performance of different references. Positive CM at time point *t* is the position weighted average of the EEG data with positive amplitude, and it is calculated with the following equations (Manjarrez et al., 2007):

(5) $$\begin{array}{l} X\left(t\right)=\frac{\sum_{a_{i}m_{i}}\left(t\right)}{\sum_{m_{i}}\left(t\right)},\;m_{i}\left(t\right)>0,i=1\ldots N\\ Y(t)=\frac{\sum_{b_{i}m_{i}}\left(t\right)}{\sum_{m_{i}}\left(t\right)},\;m_{i}\left(t\right)>0,i=1\ldots N\\ Z\left(t\right)=\frac{\sum_{c_{i}m_{i}}\left(t\right)}{\sum_{m_{i}}\left(t\right)},\;m_{i}\left(t\right)>0,i=1\ldots N \end{array}$$

where *X*(*t*), *Y*(*t*), and *Z*(*t*) are the orthogonal coordinates of CM; *a*_*i*_, *b*_*i*_ and *c*_*i*_ are the coordinates of the electrode channel *i*; and *m*_*i*_(*t*) is the positive voltage of channel *i* at time point *t*; and N is the electrode number. At any time point, a definite spatial CM point can be achieved.

### CM traveling velocity

The traveling velocity of CM at one time point was computed according to the Euclidean distance between the orthogonal coordinate of CM at one time point *t* and that at the prior time point *t-1*. These differential values characterize the propagation velocity of the ERP waveforms. The CM traveling velocity in a two-dimensional scalp field was calculated by

(6) $$\begin{array}{l} V\left(CM\left(t\right)\right)=\sqrt{{\left(X\left(t\right)-X\left(t-1\right)\right)}^{2}+{\left(Y\left(t\right)-Y\left(t-1\right)\right)}^{2}}/dt \end{array}$$

During the ERP data analysis, we normalized the electrode and CM coordinates to the head model with the sphere radius *r* = 10 cm. The sampling rate of the CM trajectory (i.e., 250 Hz in the real data analysis) was used in formula (6). Thus, we obtained the quantified CM traveling velocity with the metric (i.e., m/s).

### Simulation

A dipole source model was used for the EEG forward calculation (Yao, 2003; Yao and He, 2003). Simulation was conducted for each voxel with a discrete cubic as a dipole source. The discrete cubic grid, consisting of 1994 dipoles, was constructed and confined within radius *r* ≤ 0.86 with an inter-grid distance of 0.0905. That is, the Cartesian coordinates of the grid cubic (x, y, z) satisfy the conditions *x*^2^ + *y*^2^ + *z*^2^ ≤ 0.86^2^ and z ≥ 0. Considering that dipoles with any direction can be decomposed into three components along the X, Y, and Z directions, we studied the sensitivities of different references when dipoles were directed to the X, Y, or Z axis, respectively. The temporal process of a dipole source was simulated using a damped Gaussian function,

(7) $$\begin{array}{l} h(t_{i})=\exp\left(-{\left(2\pi f\frac{t_{i}-t_{0}}{\gamma }\right)}^{2}\right)\cos\left(2\pi f\left(t_{i}-t_{0}\right)+\alpha \right)\\ \;i=1,\cdots ,k \end{array}$$

with parameters$t_{0}=35^{*}dt,f=10Hz,\gamma =5,\alpha =\pi /2$. Forward calculation was conducted for each dipole with one direction, and then, the scalp EEG was re-referenced to the average reference (AR), linked mastoid reference (LM), CZ reference, and the neutral reference with REST. Based on the scalp distribution, CM was calculated with different references. As the forward result was the true recordings with the reference at infinity, the CM was also calculated from the original EEG distribution without any reference transformation, and regarded as the standard to evaluate the other four reference methods. The CM error between the infinity reference and any one of the four references is defined as:

(8) $$\begin{array}{l} Err=||CM_{ref}-CM_{ir}||/||CM_{ir}|| \end{array}$$

where *CM*_*ir*_ and *CM*_*ref*_ are the Euclidean norm of the CM coordinates with the reference at infinity and the other four transformed references.

### ERP application

#### Participants

Twelve healthy postgraduate students (males, right-handed, 22–27 years) participated in this experiment. All subjects gave written informed consent in accordance with the Declaration of Helsinki. The study was performed according to the guidelines approved by the Ethics Committee of the University of Electronic Science and Technology of China (UESTC). No subject reported using medication or having a personal or family history of psychiatric or neurological disease.

#### Experimental procedures and EEG recording

**Figure 1:**
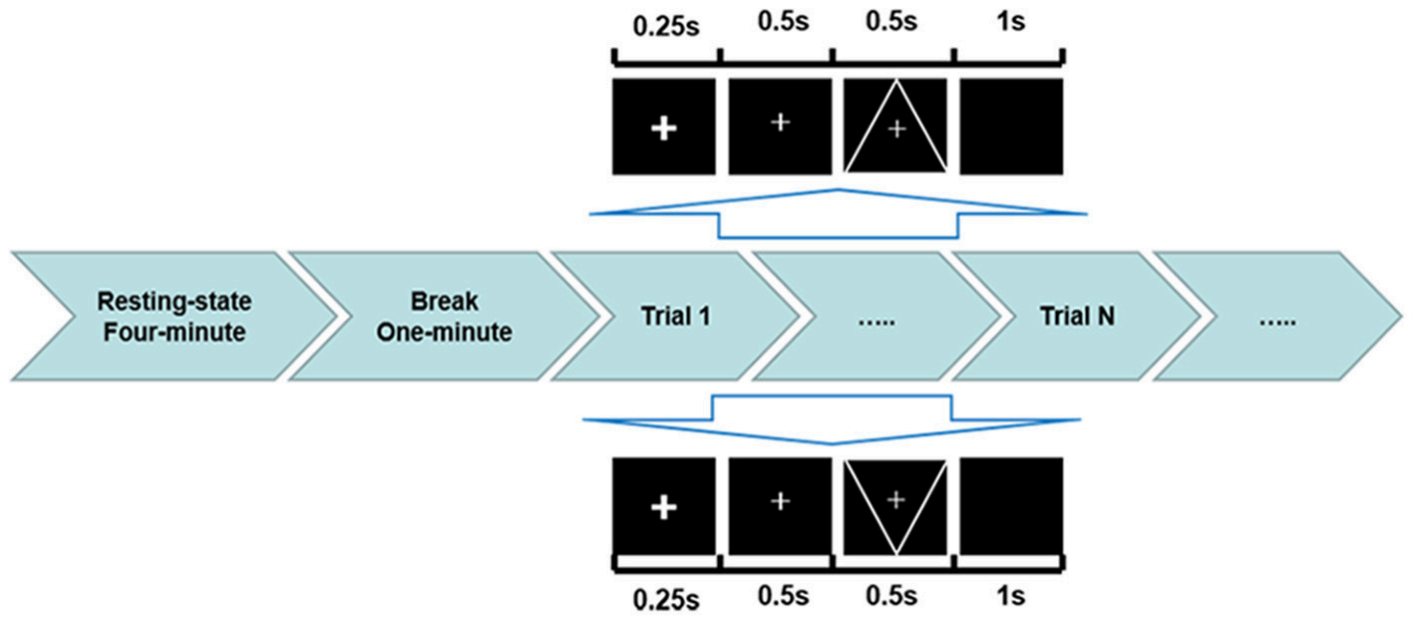
ERP experimental protocol used in the current study. Before the visual oddball task presentation, a 4-min resting state and a 1-min break were performed. In each trial, the down-oriented triangle with a thin cross in the center represented the target stimulus with an occurring probability of 20%, while the upward-oriented triangle with a thin cross in the center indicated the standard stimulus with an occurring probability of 80%.

The traditional visual oddball paradigm was implemented in this experiment. The stimulus type consisted of target stimulus and standard stimulus. The target stimulus was a downward-oriented triangle with a thin cross in the center, and the standard stimulus was an upward-oriented triangle with a thin cross in the center. Three sessions were performed, each consisting of 150 trials, occurring at probability of 80% for standard stimuli and 20% for target stimuli. The detailed procedure is illustrated in Figure 1. A 4-min resting-state EEG was initially recorded, and after a 1-min break, the P300 task was performed. During the tasks, subjects were asked to fixate on the center of the monitor. A bold cross served as a cue that appeared at the start of the task to ensure that the subjects concentrated on the monitor. After 250 ms, a thin cross lasting for 500 ms informed subjects of the stimulus onset. The stimulus was presented for 500 ms. The subjects were asked to pay attention to and count the number of target stimuli, and once the experiment was complete, they stated the number of target stimuli they counted.

EEG data were recorded by Brain Vision Recorder using a 64-channel EEG system and digitized with a sampling rate of 500 Hz (Brain Products GmbH). The EEG cap (BrainCap, EasyCap GmbH) consisted of 63 scalp electrodes distributed according to the extended 10/20 system. Electrodes Fpz, Fz, Cz, CPz, Pz, Oz were arranged along the midline of the skull, and the other electrodes were located symmetrically on both sides of the midline. The online filter band was 0.01–100 Hz and the impedance of all electrodes was maintained below 5 KΩ. The FCz and AFz electrodes served as the reference and ground, respectively. Vertical and horizontal electrooculogram (EOG) data were recorded to monitor eye movements.

#### ERP data analysis

After the EEG data has been successfully recorded, we performed the necessary pre-processing procedures using Matlab R2013a (The MathWorks Inc.). The recorded EEG data were first re-referenced to REST, AR, and LM references, respectively. CZ reference was excluded in the real data analysis because of its serious distortion to the vertex electrodes. Then, filtering (6 Hz low-pass filter; Portin et al., 2000; Li et al., 2015) was performed, and the EEG was divided into epochs with 1,000 ms (200 ms pre-stimulus and 800 ms post-stimulus). A baseline correction for the period 200 ms before stimulus onset was performed for each epoch. To discern the ocular and other artifacts, a rejection criterion of ±75 μV was used at all of the electrode sites. On average, 90% of all epochs were retained after the artifact rejection. After pre-processing, averaging of ERP epochs for the two types of stimulus was performed.

**Figure 2:**
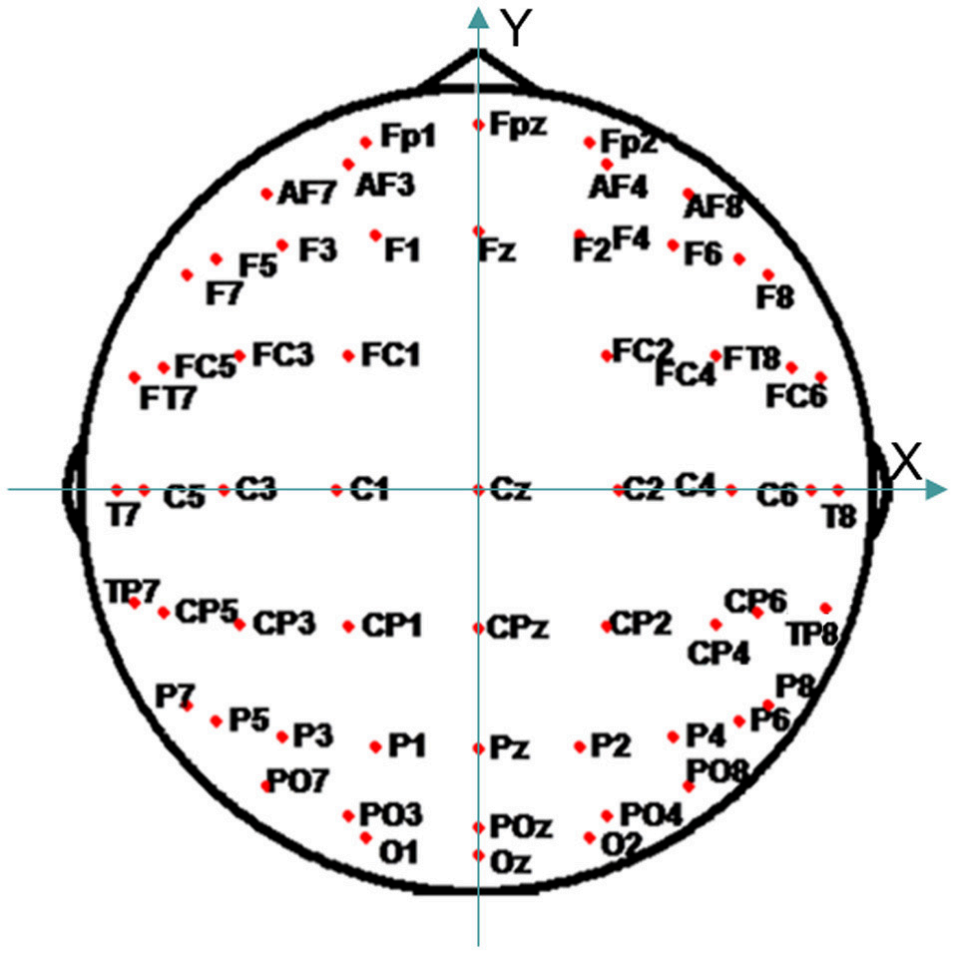
The montage of 59 electrodes used for CM analysis.

CM of the averaged ERP was then carried out with three references, REST, AR, and LM. To keep the EEG CM trajectory on the scalp, two-dimensional CM with orthogonal coordinates [*X*(*t*), *Y*(*t*)] was applied (Manjarrez et al., 2007). Figure 2 showed the montage of 59 electrodes used in ERP CM calculation, and the EOG electrodes (VEOG, HEOG) and bilateral mastoid electrodes (TP9, TP10) have been excluded.

#### Statistical analysis

One-way repeated-measures analyses of variance (ANOVAs) were performed on the values (i.e., ERP amplitude, latency, CM location, and CM traveling velocity) to evaluate the reference effects. Significant differences revealed by ANOVA were further analyzed for multiple comparisons using Tukey's *post-hoc* test. The value of epsilon (ε) of Greenhouse–Geisser would be denoted when the Greenhouse–Geisser correction was necessary. A significance level of *P* < 0.05 was used in all comparisons.

## Results

### Simulation results

**Figure 3:**
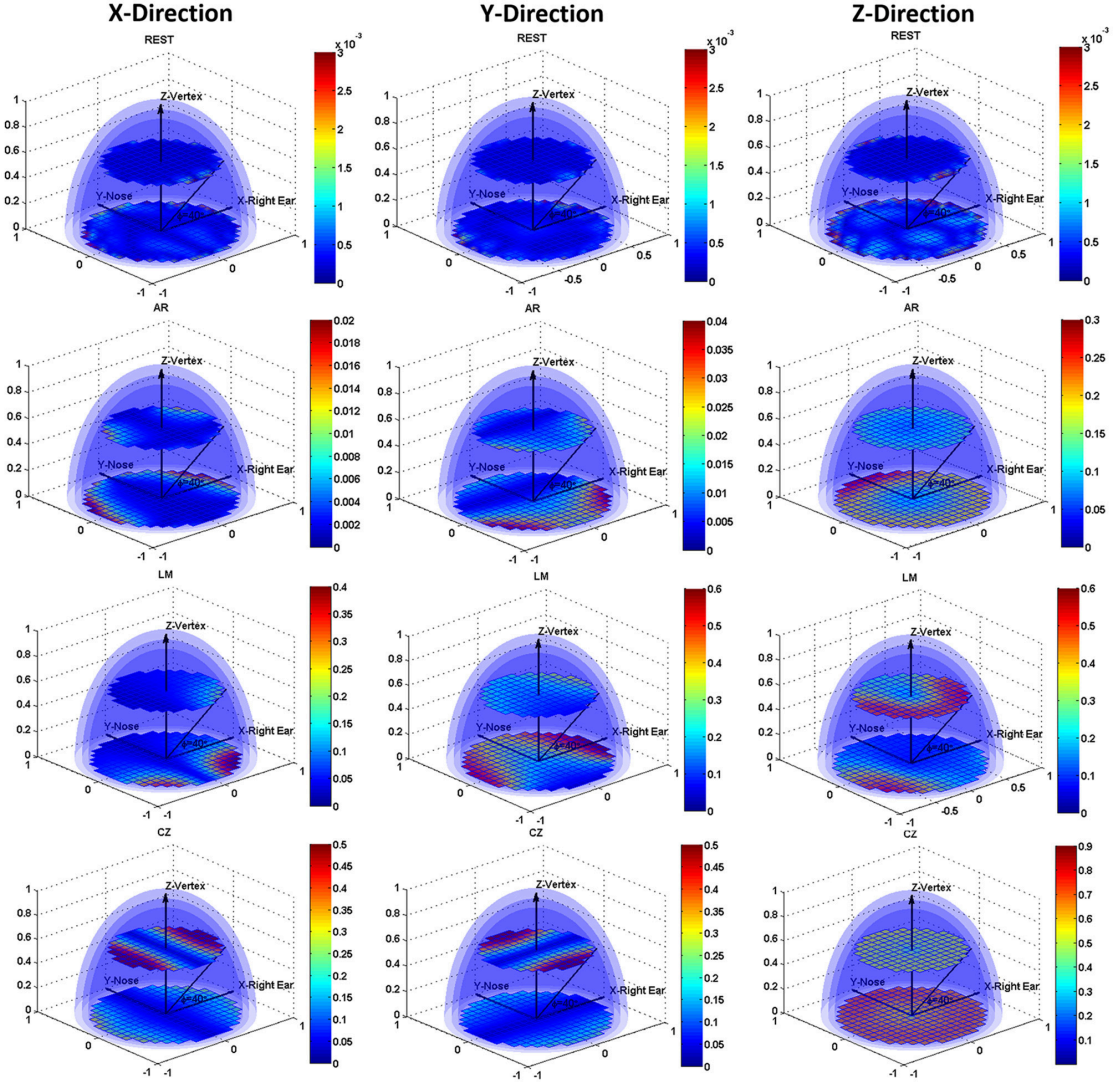
Slice display of the volume distribution for the CM errors. Errors for REST, AR, LM, and CZ references are listed in the four rows from top to bottom, respectively. The three columns from left to right represent the error maps with dipoles orientating along X-axis, Y-axis, and Z-axis.

Figure 3 shows the CM error distribution in the form of slice display, and two slices, i.e., angle with the XY plane, 0 and 40 degrees, are illustrated. The distinct CM error distribution for the different conditions is shown in the subplots. REST had the smallest error compared to the other three references. A Tukey's test revealed significant differences (*P* < 0.001) for all pair-wise comparisons among these references in any dipole direction. Furthermore, the REST reference was hardly influenced by the dipole location or direction, while the other three references had a markedly varied error distribution map as the dipole location and direction changed. When the dipoles were directed along the positive X-axis, the error focused on the edge of the positive and negative X-axis, and all references showed some degree of symmetry along the Y-axis. The AR had a larger error in the anterior areas, while the LM reference had a larger error in the posterior areas. With dipoles orientated along the Y-axis, there was still an error distribution with the symmetry along the Y-axis. For the AR, dipoles in the posterior areas had a larger error, and for the LM reference, the largest error was near the two mastoid areas. As for CZ reference, the error was aggravated in the superficial areas. When the dipoles were directed to the Z-axis, there was a larger error than in the other two directions. The AR and CZ references showed gross error in the bottom areas, and the LM reference still had the largest error in the posterior areas.

### ERP application results

#### ERP waveforms

**Figure 4:**
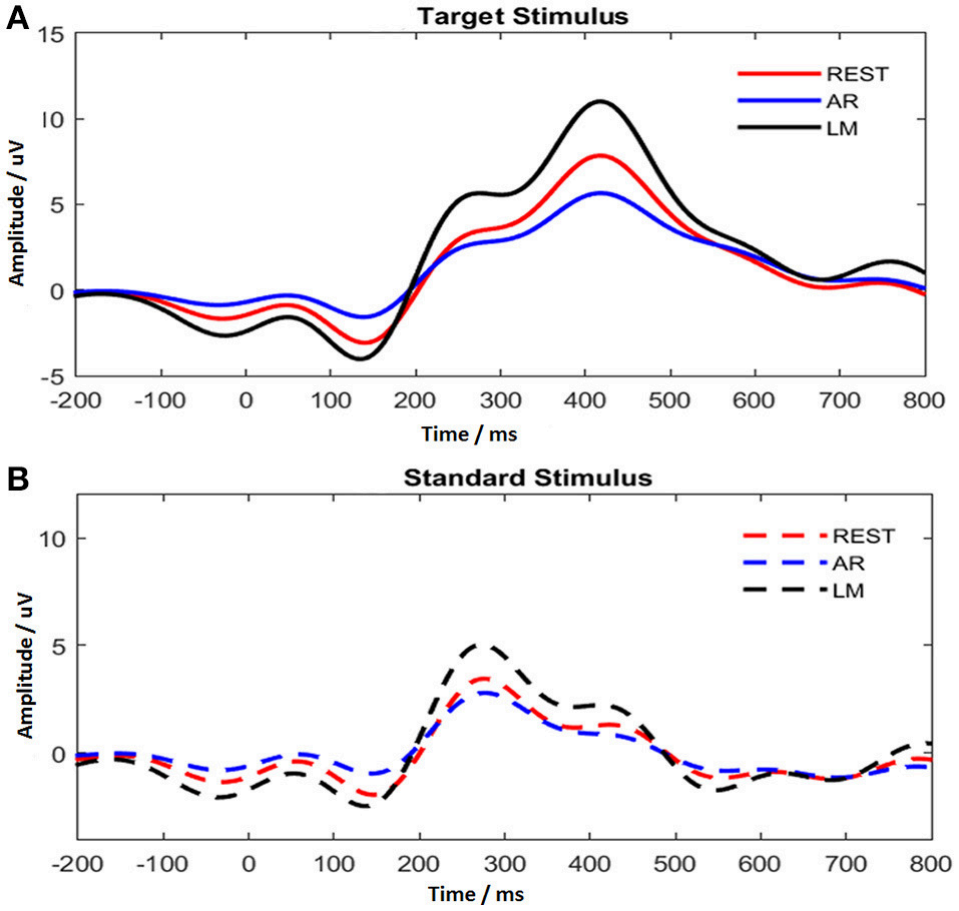
P300 time course with different references for target stimulus **(A)** and standard stimulus **(B)**.

ERPs were obtained after the data pre-processing for the three references, REST, AR, and LM. Electrodes Cz, CPz, Pz in the posterior midline of the scalp were found having the maximum P300 amplitude. To obtain a reliable estimate of P300 amplitude and latency, the averaged values across the three electrodes (Cz, CPz, and Pz) were treated as the P300 amplitude and latency for each individual subject. In Figure 4, the average ERP time course was demonstrated for two different stimuli. The target stimulus showed a prominent P300 component with long latency. The P250 enhancement was evoked by the standard stimulus, and no distinct positive component appeared after that.

In addition, ERP waveforms were altered by using different references. As shown in Figure 4, AR reduced the P300 amplitude, while LM increased the P300 amplitude. The difference for P300 amplitude with target stimulus among references was significant [*F*_(2, 22)_ = 68.251, ε = 0.541, *P* < 0.001], and pair-wise comparisons using Tukey's *post-hoc* test revealed significant differences for all comparisons (*P* < 0.001). In addition, no difference was found for P300 latency among references.

#### CM trajectory, traveling velocity, and ERP stages

**Figure 5:**
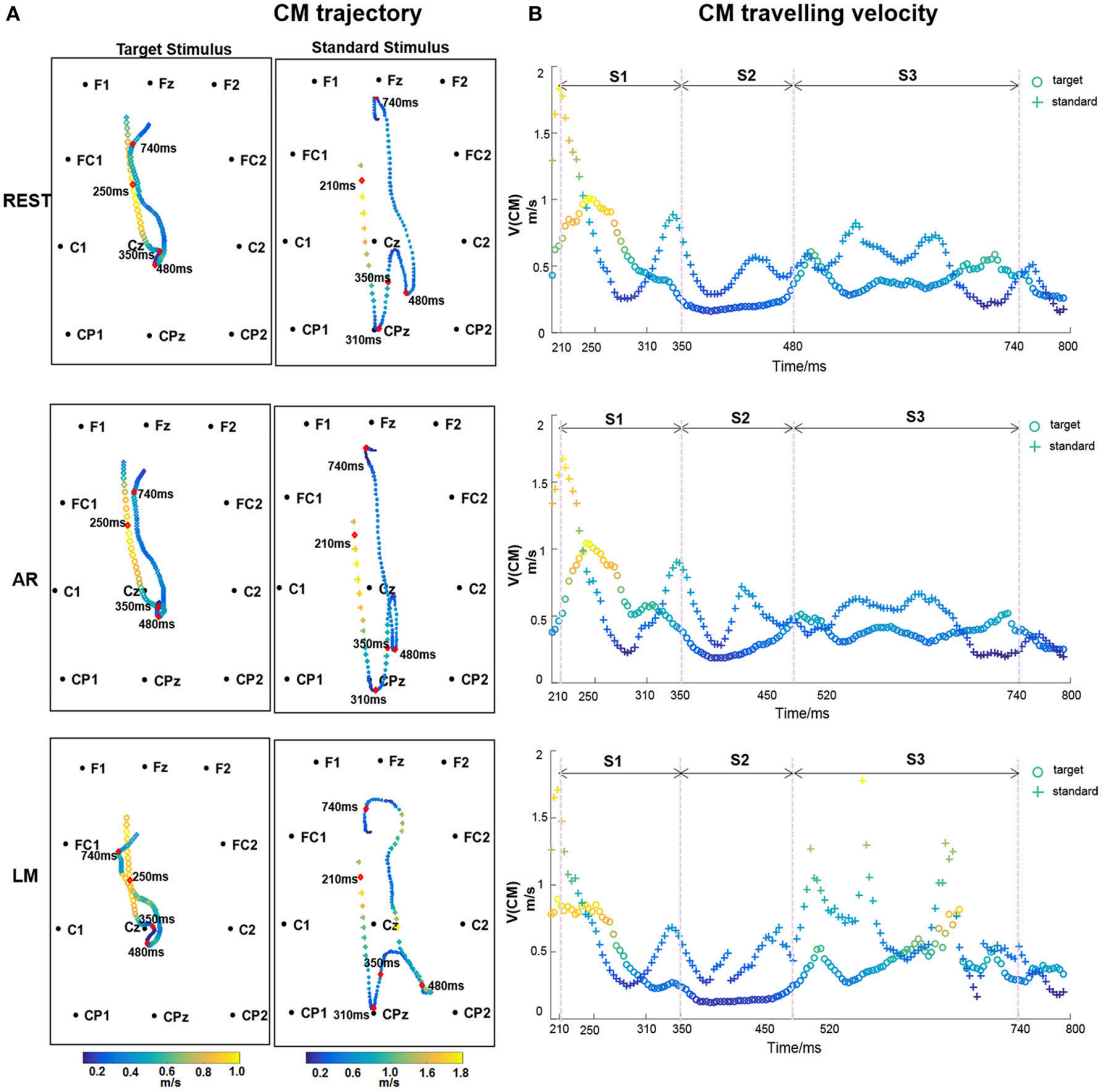
**(A)** CM trajectory of ERP with different references for target stimulus and standard stimulus. CM traveling velocity in each time point is depicted using different color, with bright color indicating the high speed and dark color indicating the low values. The time points, 210, 310, 350, 480, 740 ms after stimulus onset were labeled. **(B)** CM traveling velocity curve of ERP time course. Three stages S1 (210–350 ms), S2 (350–480 ms), and S3 (480–740 ms) are divided.

The ERP CM from 200 to 800 ms after stimulus onset was computed with one time point interval (i.e., 250 Hz sampling rate). The CM trajectory constituted 150 points. As shown in Figure 5A, the CM trajectory traveled from the frontal to parietal areas and then back. However, the characteristic CM trajectory stemmed from different stimuli. For the target stimulus, the ERP CM shifted from the frontal area to the vertex near the Cz electrode at approximately 350 ms and then extended a short distance along the middle line. After a short period, the CM of the target stimulus returned to the frontal area. For standard stimuli, the ERP CM originated from a similar frontal area and expanded its range to the parietal and occipital areas at 310 ms. After spanning a wider coverage area and circuity, the ERP CM of the standard stimulus then traveled back to the frontal areas.

Figure 5B shows the CM traveling velocity through the whole CM trajectory from 200 to 800 ms. The CM traveling velocity ranged from 0.2 to 2 m/s during the whole time course. To more intuitively show the temporal-spatial and speed change information, the normalized CM traveling velocity was mapped in the CM trajectory using different colors, with a bright color indicating the high velocity and a dark color indicating the low value (Figure 5A). For the CM traveling properties, we labeled a small number of inflection time points and divided the ERP processing into three stages: S1 (210–350 ms), S2 (350–480 ms), and S3 (480–740 ms). S1 reflected the CM traveling course from the anterior to posterior areas, corresponding to when the CM velocity dropped to the minimum. S2 was the circuitous process with lower a CM traveling velocity at the P300 stage. S3 was the return process with relatively oscillating CM velocity.

In terms of different references, distinct CM trajectory and traveling velocity were illustrated. The CM trajectory was similar between REST and AR, particularly for the S1 and S3 stages, and AR had a slight posterior shift in the S2 stage. LM expressed a distinguished trajectory. The reference effect was evaluated on the CM locations and traveling velocity with target stimulus. As for CM trajectory, one-way repeated-measures analysis of variance (ANOVAs) revealed significant differences among references for both S1 and S2 stages [S1: *F*_(2, 22)_ = 11.933, *P* < 0.001; S2: *F*_(2, 22)_ = 17.427, ε = 0.543, *P* < 0.001], and pair-wise multiple comparisons revealed significant differences (Tukey's test, *P* < 0.01) for all comparison pairs, except between REST and AR in S1 stage. Furthermore, ANOVAs and Tukey's *post-hoc* test were performed to CM traveling velocity, and similar results were obtained. Significant differences were found among references for both S1 and S2 stages [S1: *F*_(2, 22)_ = 6.840, ε = 0.667, *P* < 0.05; S2: *F*_(2, 22)_ = 9.765, ε = 0.632, *P* < 0.01], and pair-wise multiple comparisons revealed significant differences (*P* < 0.05) for all comparison pairs, except between REST and AR in S1 stage.

#### The relationships for CM traveling velocity between stimuli

**Figure 6:**
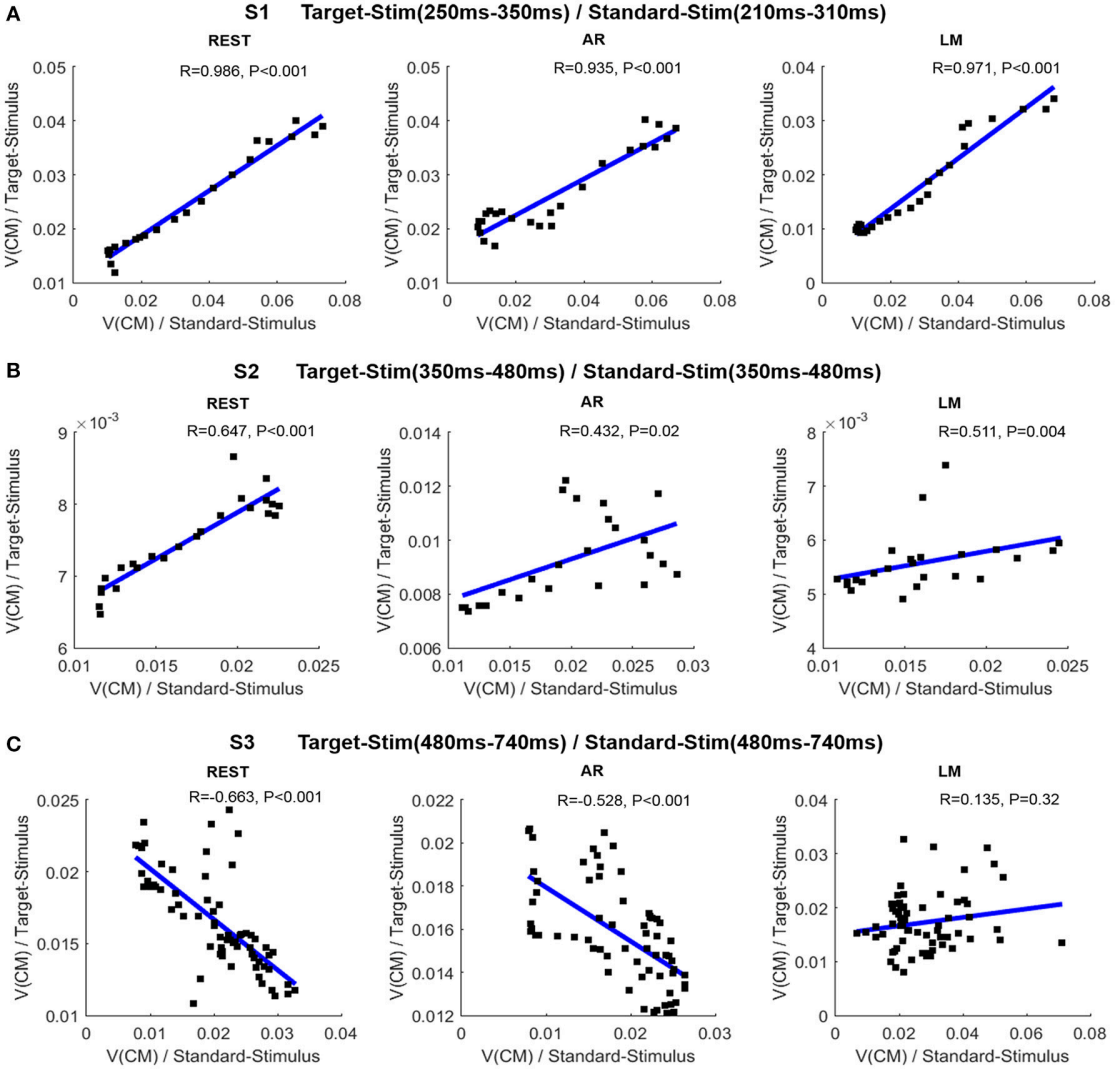
Relationship of CM traveling velocity between target stimulus and standard stimulus in different stages. **(A)** Correlations between the two conditions for S1 stage (target stimulus: 250–350 ms; standard stimulus: 210–310 ms). **(B)** Correlations between the two conditions for S2 stage (350–480 ms). **(C)** Correlations between the two conditions for S3 stage (480–740 ms). In each sub-figure, the line is the fitted line; R is the correlation coefficient, and P is the statistical significance.

The average of the CM traveling velocity across all subjects was used to investigate the correlation between the two stimulus conditions, which denoted the consistency of cognitive patterns during ERP processing. In Figure 6, the scatter points indicate time points when the CM traveling velocity was presented for the two stimulus conditions. In the S1 stage, a significant positive time-lagged correlation was shown for the target stimulus at 250–350 ms and the standard stimulus at 210–310 ms. All references shared this consistency. In the S2 stage, a significant positive correlation was revealed at 350–480 ms for REST, while the correlation was not significant for AR and LM references. Additionally, a significant negative correlation was revealed at the S3 stage for the REST and AR references. The relationship of the CM traveling velocity between the two stimuli can be better fitted linearly by REST with a higher correlation coefficient, while the other two references were more varied at certain time points.

## Discussion

### Simulation analysis

Conventional simulation verified the reference effects and takes priority over the subsequent EEG CM analysis. Errors introduced by references were influenced by the dipole location and orientation. The simulation results demonstrated that REST introduced the smallest error, while the other three references consistently distorted certain areas. The symmetrical error distribution along Y-axis of AR indicated that the effectiveness of AR depends on both the true source distribution and the assumed head surface electrode montage (Yao, 2001; Liu et al., 2015). LM reference had a profound impact on the bilateral mastoids and posterior areas which was determined by its non-zero characteristic (Tian and Yao, 2013). For CZ, the vertex areas were dramatically influenced, and this effect was aggravated in the superficial areas. Therefore, in a real ERP study, the CZ reference is always excluded because of this defect, but it can still be used as the recording reference electrode before reference transformation. This simulation may provide evidence and choice for EEG reference strategy in cases specific sources and interesting scalp areas.

### ERP processing and CM application

A majority of ERP studies used the oddball paradigm in which P300 is evoked by the infrequent target stimulus, and P300 does not appear or exhibits a smaller amplitude from the frequent standard stimulus (Kok, 1997). Figure 4 shows the classic P300 component in target stimulus and the P250 component in standard stimulus. The standard stimulus is commonly used as the background, however, intervening events related to the standard stimulus engage attention to modify the current neural representation and the additive information may be beneficial in assessing the cognitive process during the oddball task (Garcíalarrea et al., 1992; Polich, 2007). Cognitive processing in a two-stimulus oddball task is attributed to composite stages, such as, stimulus-driven attention, decision processing, and the neuronal response (Desmedt, 1980; Picton, 1992; Li et al., 2016), and P300 is thought to be a post-decision event (Desmedt, 1980). It is necessary to integrate the ERP stages to investigate the dynamic temporal-spatial patterns.

CM traveling trajectory describes the dynamic pattern of the EEG, and it is a sensitive indicator for exploring entire neural pathways in large scale cortical signals (Chao et al., 2007; Manjarrez et al., 2007) which can be extended to the positive potential, the negative potential and the power spectra application (Wackermann et al., 1993; Manjarrez et al., 2007; Qin et al., 2010). In this experiment, a two-dimensional positive CM trajectory map described the propagation of ERP waves switching from the frontal to the parietal and occipital areas and then back. This dynamic circuitous pathway produced multiple cognitive processes including attention, decision, response and post-response, probably stemming from cortico-cortical coupling. The initial CM propagation from anterior to posterior areas covered the fronto-parietal attention dominant brain regions including the frontal lobe, the center area, and part of the parietal areas (Kirino et al., 2000; Daffner et al., 2003; Li et al., 2010). The standard stimulus traveled a similar trajectory but with a wider coverage area in the parietal areas, which may indicate the more thorough cognitive processing (Garcíalarrea et al., 1992). In addition, the combination of trajectory and traveling velocity extended the observation of ERP cognitive processing. The CM traveling velocity dropped to a slower flat process with the presence of P300 comparing to other time periods. During the whole time course, the CM velocity ranged from 0.2 to 2 m/s, which was within the range of previous measurements of wave propagation velocity on the human scalp (Massimini et al., 2004; Nunez and Srinivasan, 2006b; Manjarrez et al., 2007), and it provided a quantified metric to describe wave propagation.

Three stages were divided according to the CM trajectory and velocity changes. In the earlier S1 stage, CM shifted from the frontal to the parietal area with a minimum CM speed. The time-lagged consistency of CM velocity between the two stimuli may be related with the widely appeared P250 component and the overlap of the typical positive components in both stimulus conditions, i.e., P200, P250, and earlier P3a (Garcíalarrea et al., 1992; Polich, 2007). Therefore, the cognitive treatment to different stimulus in this stage may belong to the same modality, indicating stimulus-driven process stemming from the frontal attention mechanism, involving the simulation identification, attention orienting, and cognitive evaluation (Polich, 2007). Then, CM trajectory turned back at 310 ms for the standard stimulus, and consistent cognitive modality between two stimulus conditions appeared at S2 stage starting from 350 ms. This phenomenon may suggest an additive evaluation process existing at the end of S1 due to the standard stimulus engagement. That is, after the earlier process at 310 ms, if no target stimulus was recognized, small or no P300 would appear. The following S2 indicating the P300 stage with lower CM traveling velocity may be associated with the top-down guided control and memory operation, and the positive correlation between the two stimuli showed a consistent cognitive pattern (Polich, 2007; Salmela et al., 2016). In the post-response stage at about 480–740 ms, the CM trajectory turned back to the frontal area, and the negative correlation indicated the late divergence of a cognitive pattern between two stimuli, which may be caused by the response control and mental state, i.e., mental counting and the relaxed state after responding (Strüber and Polich, 2002). An intracerebral electrode recording showed the existence of identical ERPs between the target and standard stimulus from 300 to 470 ms in most intra-cerebral sites, while a divergence was found in the late phase of the ERPs in most intra-cerebral sites after 570 ms (Kukleta et al., 2003; Damborská et al., 2012). Thus, with the help of the CM dynamic pattern, three stages were suggested for oddball visual processing: stimulus-driven orienting, top-down control and memory operation, and the post-response mental state, with the stimulus-independent and mental-dependent neural operation being dominant during the oddball tasks.

### Reference effect

The reference effect was obviously embodied on the ERP analysis, and many studies have reported that this impact varied with the temporal course and channel locations (Yao, 2001; Liu et al., 2015). In addition to the prominent waveform deflections, peak polarity reversal, and topographic shift may dramatically affect the identification, qualification and interpretation in EEG studies (Kayser and Tenke, 2010). LM, CZ, and other scalp site references that contain large potential values are undesirable in ERP studies (Kayser and Tenke, 2010; Tian and Yao, 2013). For the P300 peak, subtracting a positive average value will result in a reduction or polarity reversal for the positive potential and an increase in the negative potential, which was the cause of the topography distribution distortion and CM shift. Moreover, the distortion varied in different scalp areas. As the mastoids are located at the bilateral occipital regions, LM tends to have a greater effect on the bilateral posterior electrodes. Similarly, CZ would mess extensive areas around the vertex due to electrode location. Hence, using the non-neutral references will lead to information loss and hide the physiological nature of the condition. AR is recommended in many papers because it seems to be independent on any particular electrodes (Ferree, 2006; Nunez and Srinivasan, 2006a). However, the average of all recording channels also leads to a varying degree of effect on special locations. Comparing AR, LM, and CZ references revealed that AR produced results that were much closer to those of REST, when applied to both simulated and real ERP data. These findings indicated that AR is a better choice than LM and CZ, which have been adopted in many neurocognitive EEG studies.

REST provides a true electrically neutral reference based on the physical essence of EEG generation (Yao, 2001). It is recommended and widely used in neural cognitive and clinical applications (Yao et al., 2005; Kayser and Tenke, 2010; Qin et al., 2010; Tian and Yao, 2013; Xu et al., 2014; Liu et al., 2015). It was shown in the simulation that the error is greatly reduced with REST compared with other commonly used references. Although a realistic head model constructed for each individual subject would likely improve the accuracy of REST for each subject, previous reports (Yao, 2001; Zhai and Yao, 2005) have showed that REST is effective even when the volume conductor differs from the true head model. Comparing with other reference-free methods such as, scalp Laplacian, the cortical imaging technique (Tenke and Kayser, 2005; Nunez and Srinivasan, 2006a), the operation involved in REST is from “scalp to scalp,” therefore, the noise in data and error in head model will not be enlarged. In the current study, in terms of CM trajectory and traveling velocity, REST best represented the three stages corresponding to cognitive mechanisms. CM traveling velocity could be better linearly fitted with REST and had a higher correlation coefficient for two stimuli. This indicates that REST recovers the lost information and provides the optimal approximation for the neutral reference.

## Conclusions

In this study, the reference effect on simulated data as well as on ERP data measured by CM trajectory and CM traveling velocity was examined. The simulation results indicated that REST introduced less error than the AR, LM, and CZ references and was less affected by dipole location and orientation. As a metric to measure the dynamic pattern of EEG spatiotemporal activity, CM and the traveling velocity extend the exploration of these cognitive mechanisms. Distinct CM trajectory and CM traveling velocity for the alternative references were represented by visual oddball task processing. As REST better represented the cognitive stages, we recommend REST as a beneficial technique for pursuing the ideal zero reference.

## Author contributions

Conceived and designed the work: YQ, XX, HZ, HX, and YL. Acquired the data: FL and XX. Analyzed the data: YQ, XX, and FL. Wrote the paper: YQ and TZ. All authors revised the work for important intellectual content. All of the authors have read and approved the manuscript.

### Conflict of interest statement

The authors declare that the research was conducted in the absence of any commercial or financial relationships that could be construed as a potential conflict of interest.

## Abbreviations

REST: Reference electrode standardization technique
AR: Average reference
LM: Linked mastoids reference
CZ: Vertex reference
CM: Center of mass.
